# Mechanochemical conversion of brominated POPs into useful oxybromides: a greener approach

**DOI:** 10.1038/srep28394

**Published:** 2016-06-21

**Authors:** Giovanni Cagnetta, Han Liu, Kunlun Zhang, Jun Huang, Bin Wang, Shubo Deng, Yujue Wang, Gang Yu

**Affiliations:** 1 State Key Joint Laboratory of Environment Simulation and Pollution Control (SKJLESPC), Beijing Key Laboratory of Emerging Organic Contaminants Control (BKLEOCC), School of Environment, POPs Research Center, Tsinghua University, Beijing 100084, P. R. China; 2 Beijing Normal University, School of Environment, 19 Xinjiekouwai St., Haidian District, Beijing 100875, P. R. China

## Abstract

Brominated organic pollutants are considered of great concern for their adverse effect on human health and the environment, so an increasing number of such compounds are being classified as persistent organic pollutants (POPs). Mechanochemical destruction is a promising technology for POPs safe disposal because it can achieve their complete carbonization by solvent-free high energy ball milling at room temperature. However, a large amount of co-milling reagent usually is necessary, so a considerable volume of residue is produced. In the present study a different approach to POPs mechanochemical destruction is proposed. Employing stoichiometric quantities of Bi_2_O_3_ or La_2_O_3_ as co-milling reagent, brominated POPs are selectively and completely converted into their corresponding oxybromides (i.e. BiOBr and LaOBr), which possess very peculiar properties and can be used for some actual and many more potential applications. In this way, bromine is beneficially reused in the final product, while POPs carbon skeleton is safely destroyed to amorphous carbon. Moreover, mechanochemical destruction is employed in a greener and more sustainable manner.

In 2004, a global Convention was ratified in Stockholm to outlaw twelve chemical substances that “remain in the environment, are transported over large distances, bioaccumulate through the food web, and pose a risk of causing adverse effects to the environment and human health”^1^, defined persistent organic pollutants (POPs). Such pollutants, called the “dirty dozen”, are chlorinated organic compounds, which include intentionally produced chemicals that had been employed in disease control, agriculture, and industry; and unintentionally generated molecules, mainly by-products of human activities. The presence of chlorine atoms in all dirty dozen compounds stands out, so POPs’ adverse effects have been ascribed to this element.

However in the last decade, evidences for toxicity and potential carcinogenicity of brominated organic compounds have been accumulating, as well as for their persistency into the environment^2^,^3^,^4^,^5^,^6^,^7^. These compounds, due to their physicochemical properties and chemical stability, are chiefly used as flame retardants in a large variety of products (e.g. polymers, electric devices, construction materials, and textiles). Brominated flame retardants (BFRs) can enter the environment through multiple pathways: emissions during manufacturing, from products during use, and from combustion and leaching from landfills or recycling when BFR containing material are disposed^8^,^9^,^10^,^11^. Hence an increasing number of BFRs are being classified as POPs and listed for ban by the Stockholm Convention^12^: Hexabromobiphenyl and two commercial mixtures of polybrominated diphenyl ethers (PBDEs), one with tetra and pentabrominated congeners and another one with hexa and hepta congeners, were listed in 2009; hexabromocyclododecane (HBCDD) was added in 2013. DecaBDE was also proposed in 2013 for inclusion in the Convention as it was considered to meet the screening criteria for persistence, bioaccumulation, long-range transport and adverse effects^13^. Other compounds such as tetrabromobisphenyl A (TBBPA) and hexabromobenzene are accumulating proofs about their toxicity^14^,^15^,^16^, thus such chemicals have a great chance to be proscribed in the next years.

Currently, an inventory of obsolete organobrominated chemicals (i.e. already manufactured but prohibited from use) is not available. It was estimated that the world annual production of PBDEs was 67,000 metric tons per year in 2001^17^, while HBCDD production rate before 2013 was estimated to be 28,000 tons per year^18^. Hence, it is plausible that huge amounts of obsolete BFRs are stockpiled in the world.

Such toxic waste deserves adequate disposal in an environmentally sound manner. High temperature incineration is the first candidate technology because it is largely available on the market^19^. However, two issues dissuade its employment: the generation of HBr, which can corrode the metallic structure and the refractory material of the incinerators, remarkably increasing maintenance costs; and the sensible risk of unintentional formation of brominated dioxin-like compounds^20^. In addition, incineration would not allow cheap recycle of a valuable element such as bromine^21^, whose price since 2008 has a growing tendency, due to the expanding markets and major increases in energy costs, raw materials, regulatory compliance, and transportation^22^. A novel process that can assure the safe disposal of obsolete BFRs (i.e. achieving the complete destruction of molecular structure, but averting the generation of toxic by-products) and permits the beneficial reuse of bromine is required.

Mechanochemistry (MC) is a fast developing research area for new materials preparation. Utilizing special high energy mills, it is possible to carry out MC transformations and reactions to produce a large variety of materials with improved peculiar properties that cannot be prepared by conventional methods^23^,^24^,^25^. Moreover, MC procedures are green: Reactions are carried out at solid state, so solvent regeneration/disposal is not necessary, and processes in milling facilities are simpler than in conventional plants^26^,^27^.

Over the last two decades high energy ball milling (HEBM) has been also utilized to destroy effectively a large variety of pollutants, included POPs^28^. It has been demonstrated that halogenated organic pollutants can be mineralized to halides and amorphous carbon within few hours^29^,^30^,^31^,^32^. However MC destruction is usually conducted with large excess of reagent (often a zero valent metal or a metal oxide, e.g. Fe^0^ or CaO) to ensure complete destruction. In general, the milling residue is mostly constituted by unreacted reagent with a certain percentage of metal halide and carbonaceous matter, even after several re-utilization cycles^33^. A new and greener approach is necessary to make this technology economically viable and environmentally sustainable.

In our previous study, DecaBDE was succeeded to be destroyed in a planetary ball mill, utilizing bismuth (III) oxide as co-milling reagent at stoichiometric ratio^34^. The pollutant was completely converted into amorphous carbon (and carbon oxides as well), while Bi_2_O_3_ was selectively and entirely transformed into oxybromide (BiOBr). The powder product was beneficially used to photo-degrade methyl orange (employed as model pollutant) under visible light in water, due to the remarkable photocatalytic properties of BiOBr^35^.

The results of this initial investigation comforted the idea that HEBM can be used more proficiently than for mere POPs destruction. It can convert pollutants into useful products in a cost-effective manner, but assuring at the same time their safe disposal. In order to corroborate such idea, a wider investigation is carried out in the present work. Expressly, we demonstrate that three objectives can be achieved adopting a novel and more environmentally friendly approach to MC destruction.

First, to perform the safe and cheap MC destruction of a larger number of different brominated POPs. Specifically, four BFRs of major concern today are employed and their carbon skeleton is entirely converted into inorganic carbon. Hence the hazardousness is eliminated.

Second, to produce oxybromides of potential interest with notable characteristics, i.e. BiOBr and LaOBr, from different oxides and bromine donors (i.e. BFRs). Bismuth oxyhalides (in particular BiOBr and BiOI) have shown remarkable photocatalytic properties^36^,^37^. Their layered structure with an internal static electric field perpendicular to each layer can induce an effective hole-electron separation, thus inducing in water high production rate of radical species that are responsible for pollutants degradation^35^. Lanthanum (and lanthanides) oxyhalides are promising materials with unique luminescent, catalytic and electric properties. Since three decades, LaOBr is utilized as X-ray radiographic receptor in intensifying screens for medical imaging; such technology improves diagnostic performance and reduces X-ray exposure^38^. Besides, of particular interest is the potential use of lanthanide oxyhalides as chemically stable and low-phonon energy hosts for doping of optically lanthanide ions in lighting applications^39^.

Third, to comply with a greater number of Green Chemistry principles^40^, thus improving the sustainability of the MC destruction technology.

## Results

### Applicability of various brominated pollutants - reaction with Bi_2_O_3_

**Figure 1:**
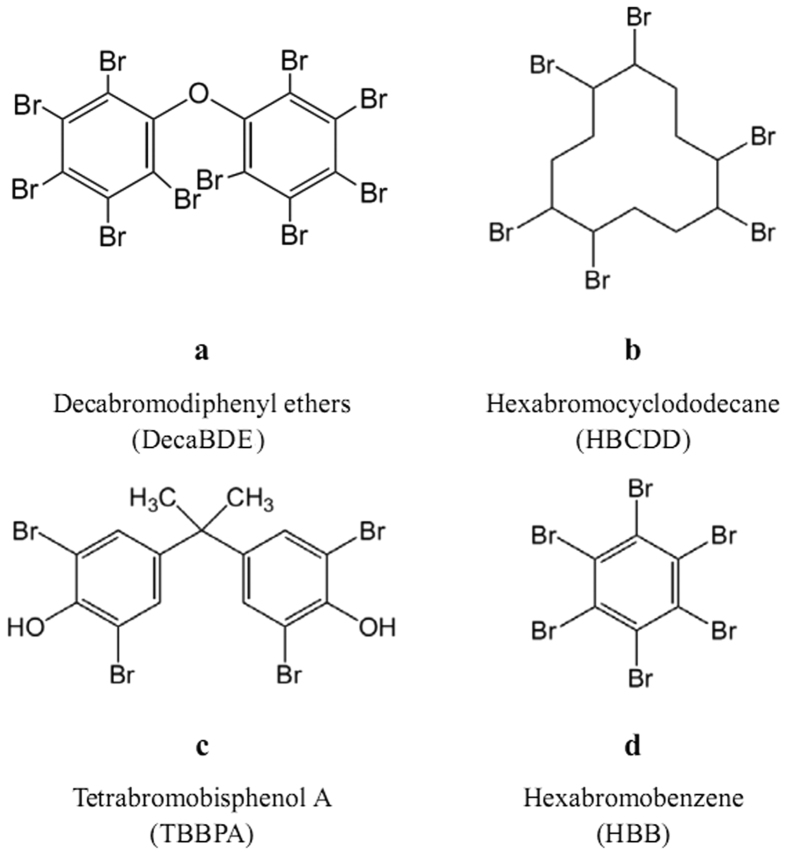
Chemical structures of the four BFRs used as bromine donors for the oxybromide formation reaction.

Bismuth oxide was tested with various brominated pollutants to verify the effectiveness of MC destruction, as well as to prove their potential employment as bromine source for the MC production of oxybromide. Four BFRs were chosen for the experiments: Decabromodiphenyl ether (decaBDE, Fig. 1a), hexabromocyclododecane (HBCDD, Fig. 1b), tetrabromobisphenol A (TBBPA, Fig. 1c), and hexabromobenzene (HBB, Fig. 1d).

**Figure 2:**
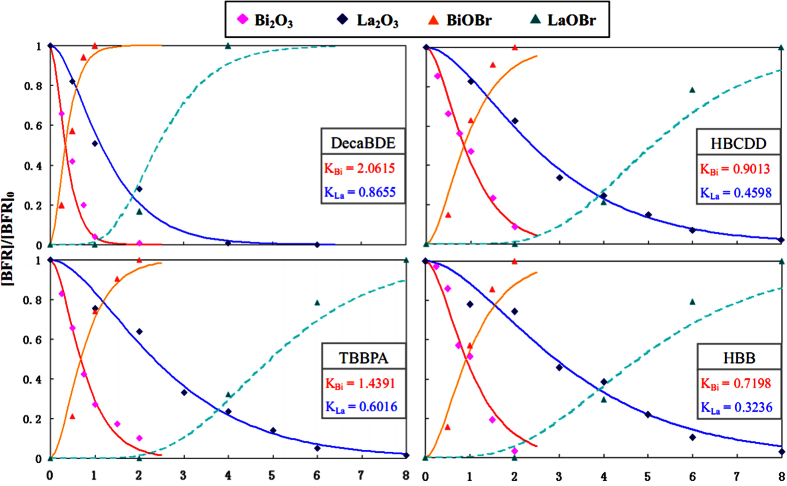
MC destruction trends of the four BFRs during ball milling with Bi_2_O_3_ or La_2_O_3_ as co-milling reagent and their corresponding product (i.e. BiOBr and LaOBr, respectively).

Results of HEBM experiments indicate that the good performance achieved in the preliminary work with decaBDE^34^ is confirmed also with the other BFRs (Fig. 2). The four brominated compounds are destroyed after 2 h ball milling utilizing a Bi:Br molar ratio of 1.

A kinetic model, which was already employed to describe MC degradation of chlorinated pollutants^41^, can be used to evaluate kinetic constants K and assess BFRs reactivity:
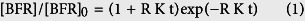
where [BFR]/[BFR]_0_ is the remaining amount of the brominated compound after a milling time of t over its initial quantity, and R is the reagent ratio (i.e. oxide-to-BFR weight ratio). Straight lines in Fig. 2 represent the model fitting of experimental data, and K values are written therein.

Kinetic constants indicate that the reaction rate of BFRs destruction has the following order:
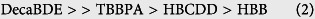


Strictly speaking, this trend reflects the degradability of BFR molecules under the mechanical action, which causes their fragmentation. DecaBDE has a weak point in the oxygen bridge that can be easily broken during milling. Analogously, the dimethyl-methenylic bridge in TBBPA represents a probable first rupture point for this molecule. The cyclododecane ring of HBCDD appears to be a quite stable structure, but not more stable than the aromatic ring of HBB. However, these considerations are not supported by experimental results. Indeed, intermediate compounds of the MC reaction were not found, because they are often short-lived radical species that are difficult to detect^42^,^43^.

Generally speaking, the final products of halogenated POPs MC destruction are halides and carbonaceous matter. Quantification of free bromides (according to the methodology described by Zhang *et al*.^44^) highlights a very low concentration of this specie after the MC reaction: Less than 5% of the theoretical amount was detected. This result suggests that bromides are incorporated by HEBM into Bi_2_O_3_ to form a water-insoluble product, i.e. bismuth oxybromide, and that such process is rather quick.

**Figure 3:**
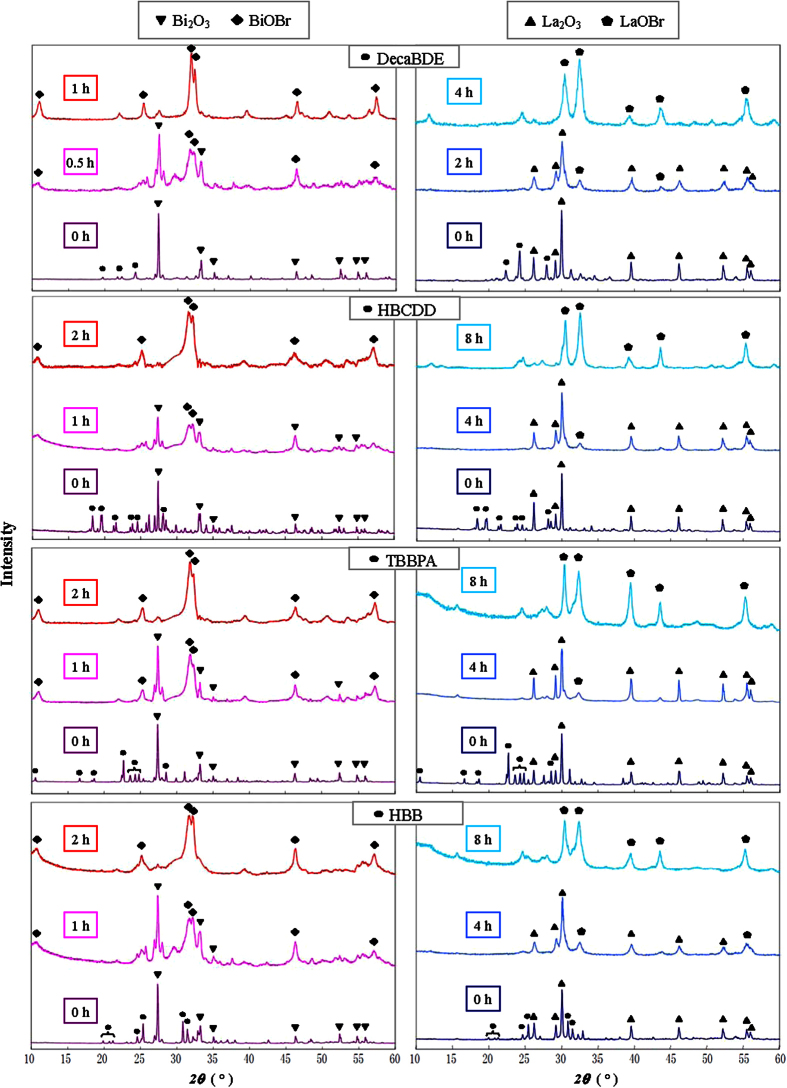
XRD spectra of the four BFRs milled with Bi_2_O_3_ or La_2_O_3_ at various milling times.

X-ray diffractometry (XRD) analyses confirm the formation of BiOBr during milling when all four BFRs are utilized as bromine source (Fig. 3). The diffractograms of unmilled samples are characterized by the presence of Bi_2_O_3_ (whose representative peak is placed at 2θ  = 27°) and flame retardants. With the HEBM ongoing, BFR patterns disappear soon, due to the quick chemical and structural changes determined by milling, while relic peaks of the oxide and new peaks of the oxybromide are visible (BiOBr has a typical close doublet at 31.8° and 32.3°). At the longest milling time, only the oxybromide’s peaks are present in the diffractogram, demonstrating the near-100% conversion of the oxide into the product of interest. The weight percentage of BiOBr at different milling times was calculated employing a web tool for Rietveld fitting of XRD patterns^45^ (Fig. 2). The straight lines represent the product formation according to the above mentioned model (with the same K value); it satisfactorily fits experimental data for all BFRs. Hence, the destruction occurs without any intermediate rate-limiting steps and the reactivity trend (eq. 2) really depicts the pollutants destructibility. In other words, the conversion of BFRs corresponds to the conversion of BiOBr. Moreover, the solid state reaction occurs independently from the bromine source and with a stoichiometric formation of the product.

Thermogravimetric analysis (TGA) is useful to quantify both inorganic and organic fractions in the product during milling. In fact, the four BFRs (as the majority of organic compounds) are thermolabile and, under nitrogen flow and above the decomposition temperature, are transformed into volatile low molecular weight compounds, gaseous HBr, and char^46^. Besides, the amorphous and graphitic carbon generated by HEBM of BFRs are quite stable under nitrogen, even at high temperatures^47^.

**Figure 4:**
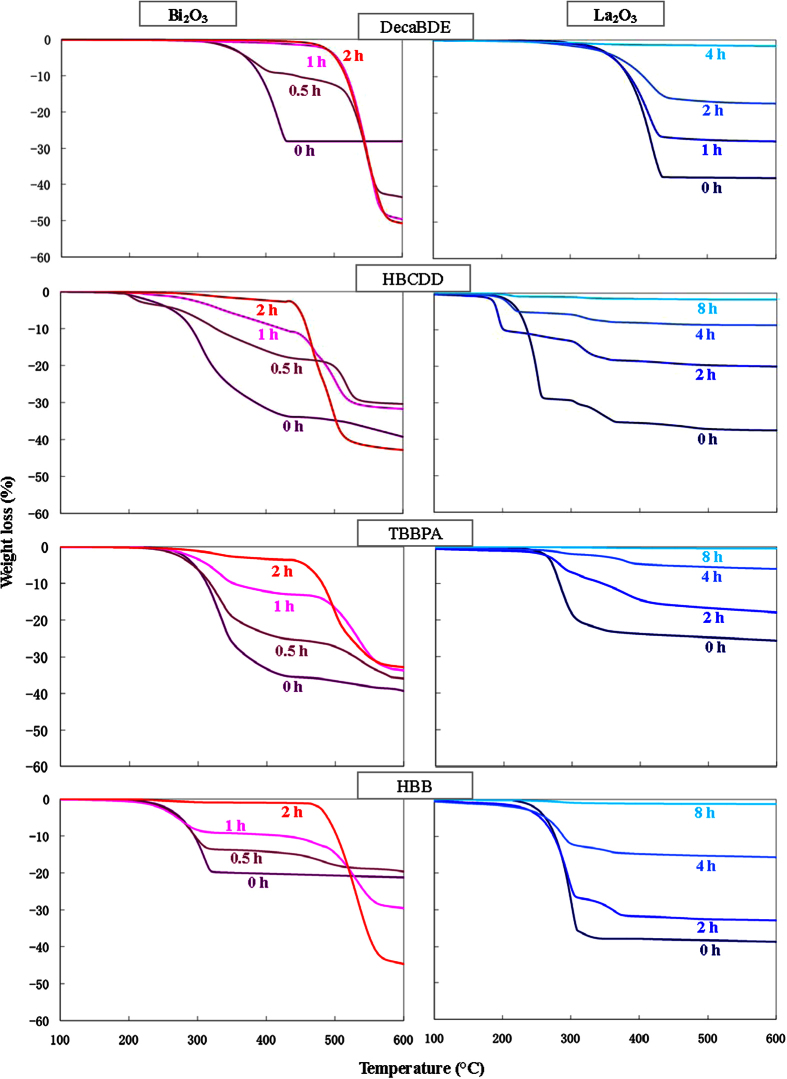
Thermograms of the four BFRs milled with Bi_2_O_3_ or La_2_O_3_ at various milling times.

Thermograms (Fig. 4) show a transition at 200–300 °C for all unmilled samples, which is due to the decomposition of BFRs, each one with its specific starting temperature. For the milled specimens, the weight loss at the 200–300 °C transition decreases with the milling duration, suggesting that the products of flame retardants MC destruction are not organic fragments but inorganic amorphous and graphitic carbon. Theoretical weight losses are consistent with experimental data (Table S1 of Supplementary Information).

Bismuth oxide is thermally stable within the scanned temperature range, so no further transitions are observed in unmilled specimens. On contrary, bismuth oxybromide above 500 °C is decomposed into Bi_2_O_3_ and the volatile gas BiBr_3_^34^, so a weight decrease is observed in milled samples due to the following reaction:
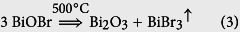


Such decomposition causes the theoretical loss of nearly 50% of oxybromide’s weight, and experimental data agree satisfactorily with calculated values (Table S1).

In order to ascertain the chemical transformation undergone by the four BFRs and whether they are completely destroyed to generate a non-hazardous residue, Fourier transform infrared (FT-IR) and Raman spectrometric analysis were carried out on samples.

**Figure 5:**
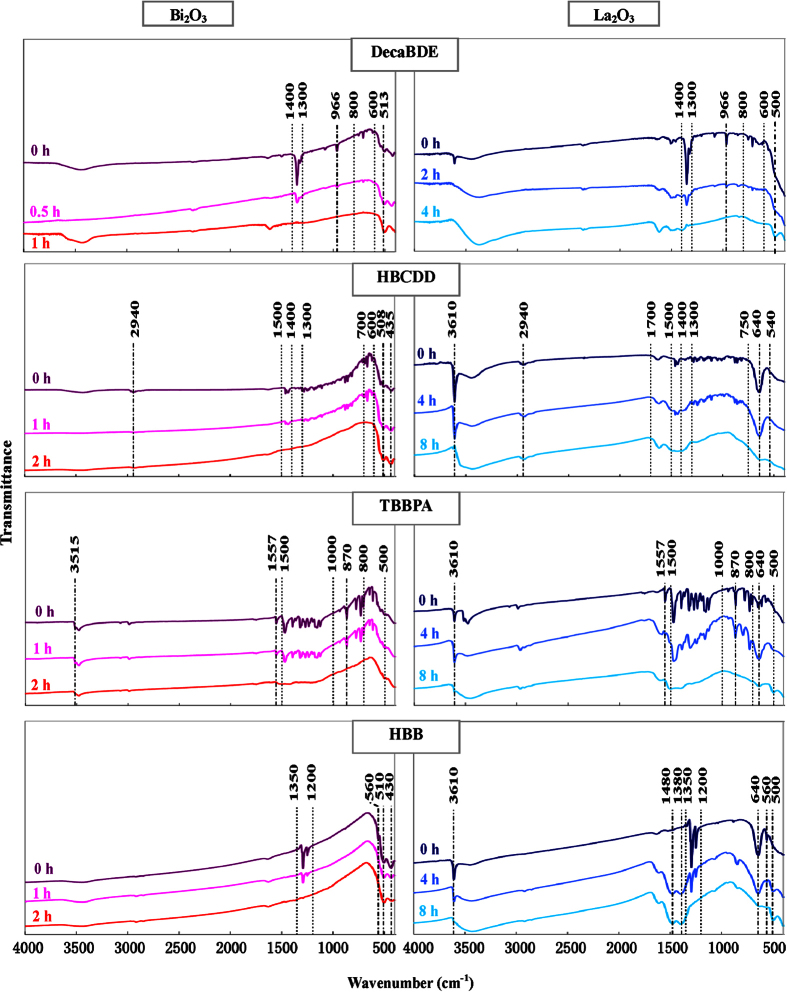
FT-IR spectra of the four BFRs milled with Bi_2_O_3_ or La_2_O_3_ at various milling times.

The FT-IR spectra (Fig. 5) of the initial mixture show unequivocally the presence of the BFR: Generally speaking, the absorption bands in the 700–1500 cm^−1^ wavenumber range can be attributed to the BFRs’ carbon skeleton vibration modes; furthermore, a typical band, roughly from 500 to 800 cm^−1^, is due to vibrations of C–Br bonds (characteristic absorption bands of each compound are listed in Table S2 of Supplementary Information). During milling such representative peaks and bands for each molecule disappear, indicating a complete destruction of the BFR’s carbon skeletons, as well as the rupture of C–Br bonds.

With regard to Bi_2_O_3_, it shows two characteristic peaks at 510 cm^−1^ and 430 cm^−1^, ascribed to Bi–O stretching modes^48^,^49^. However, these peaks are useless to distinguish the oxide from the oxybromide, being present in both compounds, thus no relevant change in the two signals is detected.

**Figure 6:**
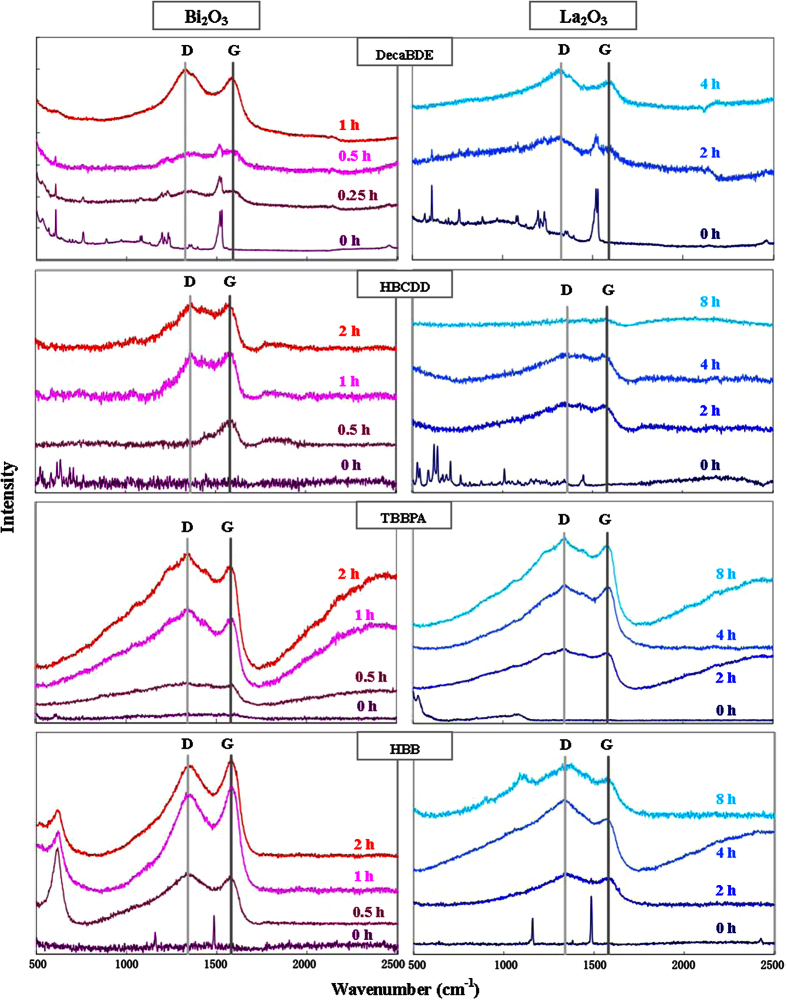
Raman spectra of the four BFRs milled with Bi_2_O_3_ or La_2_O_3_ at various milling times.

Raman spectra are consistent with FT-IR results (Fig. 6). For long milling times, all samples show the presence of two bands, called “D-band” (1330–1380 cm^−1^) and “G-band” (1540–1580 cm^−1^), which are attributed to the presence of carbon. In particular, D-band is attributed to sp^2^-bonded carbon with a disordered (i.e. amorphous) structure, while G-band is originated by graphite. In agreement with a large literature, the main final product of the HEBM of organic pollutants is amorphous and graphitic carbon^29^,^31^,^33^,^34^,^50^. It is worth reminding that stoichiometric reagent ratio was used to prepare the reaction mixture: Under such condition, complete destruction of halogenated organic pollutants (with other co-milling reagents) was very rarely achieved by HEBM^28^. Following a radical mechanism, the carbon atoms firstly form stable aromatic rings, thus generating a graphitic structure^42^,^43^; secondly, further intensive milling destroys the ordered structure producing amorphous carbon^32^. Raman spectra are consistent with such finding: For longer milling times the D-band is slightly more intense than the G-band, and, conversely, some samples milled for shorter times show the rise of the sole G-band (particularly evident in Fig. 5 for HBCDD co-milled with Bi_2_O_3_ for 0.5 h).

Significant amounts of CO_2_ were found in the jar atmosphere after milling (Table 1), in agreement with our previous result^34^. Carbon dioxide percentages vary with the BFR, suggesting that the oxidation reaction is related to the molecular structure and its degradation mechanism.

Carbonization seems to be the sole aspect that presents significant diversity among the four tested BFRs. D- and G-band of TBBPA and HBB in Raman spectra are sharper than those of DecaBDE and HBCDD, implying a higher carbonization degree. On the other hand, DecaBDE and HBCDD produce larger amounts of CO_2_ during the HEBM, in comparison with TBBPA and HBB.

As a matter of fact, the presence of carbonaceous matter represents an impurity in BiOBr and may be a problem for industrial employment of the produced material, but mild temperature calcination under air can remove such secondary product, assuring the oxybromide purification. On the other hand, recent findings suggest that the presence of carbonaceous material can extend the adsorption edge toward the visible region in some photocatalysts^51^,^52^. It cannot be excluded that carbonaceous impurity can be utilized (as-generated or after apposite treatment) to enhance BiOBr photocatalytic efficiency.

### Applicability of different oxides - reaction with La_2_O_3_

Lanthanum (III) oxide can reproduce the same solid state reaction of Bi_2_O_3_ under HEBM conditions, and LaOBr is the final product.

Experimental results point out that La_2_O_3_ determines a slower destruction rate and requires 8 h milling to achieve the entire destruction of all BFRs, except for decaBDE that necessitates of only 4 h (Fig. 2).

Comparison of kinetic constants indicates that bismuth oxide is approximately 2 times faster than La_2_O_3_. Such finding is coherent with previous results, which denoted a superior destruction rate of Bi_2_O_3_ compared to calcium oxide^34^, a widely employed co-milling reagent for many halogenated compounds^28^.

Also in this case, concentrations of free bromides leachable by water were very low (<5% of the theoretical amount).

XRD spectrograms are congruent with the results obtained with Bi_2_O_3_ (Fig. 3). BFRs patterns, clearly detected in unmilled samples, disappear in milled ones; La_2_O_3_ (with a characteristic doublet at 29° and 30°) is still observed at the intermediate time of milling, while new peaks of LaOBr (which can be represented by a doublet at 30° and 32°) are also visible. At the longest milling time, only the oxybromide is found, thus confirming that it is the sole main product of the MC reaction. However, products quantification by Rietveld refinement highlights a slower formation rate compared with BFR degradation rate (Fig. 2; dashed lines depict generic sigmoid trends that fit experimental data), so the BFR conversion at a certain milling time does not match LaOBr formation conversion. Therefore, after the initial fragmentation, pollutants undergo a slower destruction in comparison to the reaction with Bi_2_O_3_. With regard to destructibility of each BFR, the following order is observed:
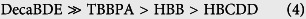


Concerning TGA, the most relevant difference with Bi_2_O_3_ results is that LaOBr is not thermally decomposed in the scanned temperature range. Therefore, only weight losses caused by BFRs thermal decomposition are detected (Fig. 4); experimental data are in good agreement with theoretical values (Table S1).

The consistency between MC reactions with Bi_2_O_3_ and La_2_O_3_ is reflected also in the fate of BFRs during milling.

FT-IR analysis confirms the total destruction of brominated compounds (whose characteristic absorption bands are reported in Table S2) during the MC reaction, and accidental organic by-products are not detected (Fig. 5). Lanthanum oxide is characterized by a peak near 500 cm^−1 ^53, but such peak is not useful to distinguish the oxide from its corresponding oxybromide. La_2_O_3_ has a notable tendency to absorb humidity and CO_2_ to form hydroxide and carbonate, respectively. In fact, all specimens with La_2_O_3_ (except samples with decaBDE, which were analyzed soon after the HEBM) present two visible peaks attributed to the formation of La(OH)_3_. Specifically, the peaks near 640 and 3610 cm^−1^ can be ascribed to La–O–H bending and O–H stretching modes of La(OH)_3_ respectively. These peaks disappear in the specimen with LaOBr (i.e. at the longest milling time) because such compound does not react with atmospheric humidity. This fact, together with the presence of La–O vibration peak (500 cm^−1^), can be considered an additional proof of the LaOBr formation during HEBM. On contrary, carbonate (whose band is characterized by two peaks at 1380 and 1480 cm^−1^) is a by-product of the destruction reaction: It is detected in all milled samples and is produced by combination of the CO_2_ deriving from BFRs destruction with lanthanum compounds. Reasonably, the low percentages of carbon dioxide observed in the jar gas phase after HEBM (Table 1) are caused by such reaction.

Apart from carbon dioxide, the final products of BFR destruction are amorphous and graphitic carbon, as corroborated by Raman spectra (Fig. 6). The D- and G-band are present in the spectra of all milled samples. Similarly to the results with Bi_2_O_3_, spectra indicate a different degree of carbonization, which is higher for TBBPA and HBB, and lower for decaBDE and HBCDD.

### Reaction mechanism

Analytical outcomes are in substantial agreement with previous results reported in literature for MC degradation of halogenated compounds. This fact suggests that the MC reaction between organobrominated compounds and metal oxides very likely follows the known pathway.

Generally speaking, during HEBM the number of crystal defects in solids increases rapidly, producing peculiar physicochemical characteristics in the milled material, included a higher chemical reactivity^23^,^26^,^54^. Such phenomenon, called “mechanical activation”, very likely happens also during MC degradation of organic compounds^28^.

Qiwu Zhang and co-workers^55^ investigated the activation of CaO and its reactivity toward chlorobenzene. They deemed the oxide anion in CaO lattice to be induced by the mechanical energy provided by ball hits to free electrons on crystal surface. This mechanical activation of CaO is the first step of the solid state reaction with halogenated organic molecules and it reasonably also happens employing other oxides.

**Figure 7:**
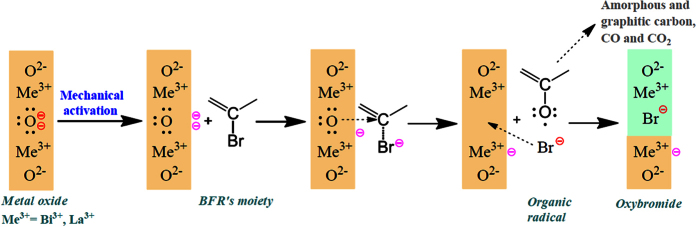
Proposed mechanism for the oxybromide formation reaction under HEBM conditions.

According to the results from Qiwu Zhang’s research group^29^,^55^,^56^, the following reaction mechanism of BFRs can be proposed (Fig. 7). After the metal oxide is mechanically activated, a carbon atom of a BFR molecule captures an electron doublet from the O^2−^ centres on the metal oxide’s surface, thus creating a C–O bond and inducing a charge transfer to the carbon. If the carbon is bound to a bromine atom, the latter enhances carbon’s electrophily, which captures more easily the electrons. Soon after, the negative charge is transferred from carbon to the bromine atom, so this latter detaches from the molecule as bromide. Bromides are trapped into the (Bi,La)_2_O_3_ lattice; the residual organic fragment remains bound to the oxygen as radical, while a free electron is left on the metal oxide surface for further reaction. The observed higher reactivity of decaBDE compared to the other BFRs may be due to its higher bromination degree, which implies a superior chance of radical formation. The oxygen of the radical, which is still lacking one electron, brings it back inducing further collapse of the BFR molecule’s carbon skeleton. Specifically, the oxygen can restore its electronic octet taking electrons from carbon, thus forming CO_2_ (and small amounts of CO^34^), or dehydrogenating the BFR to generate water and amorphous/graphitic carbon^42^,^50^. The latter pathway seems to be more relevant in TBBPA and HBB destruction.

Carbon dioxide formation may be important to determine oxides reactivity. CO_2_ is released in the jar headspace when Bi_2_O_3_ is employed as co-milling reagent, namely this gas is not trapped into reagent’s lattice and does not interfere with the oxide’s reactivity. On contrary, carbon dioxide is incorporated by La_2_O_3_ and LaOBr to form La_2_(CO_3_)_3_ and La(CO_3_)Br respectively. Analogous products were observed by Lee *et al*. after the mechanochemical reaction between La_2_O_3_ and polytetrafluoroethylene to form LaOF and La(CO_3_)F^57^. The presence of CO_2_ in lanthanum oxide lattice may hamper the activation of the oxide ions by HEBM, thus reducing significantly the destruction rate. In this sense Bi_2_O_3_ can be considered a better reagent to achieve BFRs destruction in rapid manner. In addition, the difference in reactivity order of the four BFRs (i.e. eq. 2 for Bi_2_O_3_ and eq. 4 for La_2_O_3_) is probably due to the diverse tendency to produce CO_2_ during destruction together with the hindrance effect of this gas on La_2_O_3_. Apart from decaBDE, which shows the highest destructibility because of its remarkable bromination degree, HBCDD is the second compound that generates a significant amount of CO_2_. The lanthanum carbonate (fluoride) formation and its deleterious effect on reactivity might explain why such BFR is rather fast destroyed with Bi_2_O_3_ but shows the lowest reactivity when La_2_O_3_ is employed as co-milling reagent.

Concerning the bromides, a further specific consideration can be done. Bromides are released from the BFR molecule as a consequence of electron donation from the oxide. Oxyhalides are considered solid solutions of the metal oxide with its halide: Halogen ions occupy randomly the oxide positions in the crystal lattice^58^. The formation of the oxybromide suggests that bromides, after their detachment, are trapped into the oxide lattice due to HEBM and occupy/substitute O^2−^ nodes in the structure. Such entrapment is quite efficient because the quantity of leachable bromides by water is low (i.e. <5% of the total amount), signifying that the oxybromide formation reaction is not the rate-limiting step of the whole reaction.

The oxide centres play the key role of electron-donors, so the different reactivity of the two inorganic reagents, apart from the hindrance caused by carbonates, may be also explained by the specific surrounding environment of oxide ions in lattice. Senna^59^ proposes that during milling the changing of the coordination number of reactive centres, as well as the modification of their bond lengths and bond angles, are responsible of solids’ reactivity enhancement. Specifically, the lattice distortion and the increase of crystal defects number, in particular lattice holes (which reduce the coordination number), bring to a modification of bonds polarity because of electronic maldistribution around the nuclei that enhances polarization.

The production rate of crystal defects in the two metal oxides during HEBM (which is responsible of their chemical reactivity) is not easy to evaluate, but a good starting point might be the initial coordination number. When an ion in the crystal lattice is coordinated with few counter-ions, the formation of a single hole in the surroundings has a strong influence on its polarization. On the other hand, more numerous holes are needed to produce a significant change in electrons distribution of highly coordinated ions. Therefore, the metal oxide with the lowest coordination number for the O^2−^ should also be the most reactive.

The most common polymorph of Bi_2_O_3_ found at ambient conditions is the mineral bismite (α-Bi_2_O_3_), which crystallizes in the monoclinic P2_1_/c space group. Bismite has a peculiar structure with two Bi^3+^ sites that have different coordination number, namely 5 and 6, but the coordination number of the oxygen is 3. Lanthanum oxide has a hexagonal structure (P-3m1 space group); La^3+^ is surrounded by 7 oxides, while the O^2−^ coordination number is 6. In the previous work^34^, calcium oxide was also utilized as a touchstone to assess the performance of Bi_2_O_3_ with decaBDE as bromine source. CaO has a NaCl structure, so both Ca^2+^ and O^2−^ are coordinated with 6 counterpart ions.

These three inorganic reagents showed with decaBDE (in the present work and in the previous one) the following reactivity order (according to the observed kinetic constants: K_Bi_ = 5.01, K_Ca_ = 2.08, and K_La_ = 1.47):
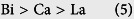
which is roughly the opposite order of the coordination number of both oxide and metal ions in the three reagents. Hence, the lower is the coordination number the higher seems to be the metal oxide reactivity. However, at this level, the proposed explanation is based on very few observations and the facility of carbonates formation during the reaction with CaO and La_2_O_3_, compared with Bi_2_O_3_ that does not originate such by-product, is a good justification as well for the reactivity trend. A larger number of metal oxides should be investigated to obtain reliable reactivity trends and figure out a plausible interpretation.

## Discussion

MC destruction of POPs is a promising technology, alternative to thermal treatment, capable to perform the safe and complete mineralization of solid hazardous waste (i.e. pure POPs or contaminated matrices). Indeed, the present work corroborates this assertion for pure pollutants: The four tested BFRs were entirely destroyed into CO_2_, graphitic and amorphous carbon, thus obtaining a complete detoxification of the milled material. In addition, BFRs’ bromine atoms were beneficially reused to produce valuable oxybromides with an actual and many more potential applications. Last but not least, results let us affirm that the proposed MC reaction represents a *greener* approach to the MC destruction technology, as addressed in detail below.

MC destruction, dealing with solid waste, possesses *per se* several characteristics that allow its classification as a green technology. First of all, it does not require any solvent, which on contrary may decrease the impact efficacy of milling bodies, thus reducing treatment effectiveness. Consequently, it complies with the Green Chemistry’ principle of “*solvents and auxiliaries reduction*”. MC destruction abides also by the principle of “*real*-*time pollution prevention*”. MC reactions necessitate of mechanical energy to occur. In general, such energy transforms the organic compounds into short-lived radical species, whose concentration decays very rapidly when the external mechanical action is not provided^60^. Hence, the reaction can be turned on and off in a simple way. This peculiarity of MC reactions allows easy monitoring of the process and facilitates the prevention of possible harmful by-products generation. In addition, powder reagents and products, although require suitable individual protection devices for workers, can be easily controlled into adequate containing structures, in agreement with the “*accident prevention*” principle.

Nevertheless, some aspects of MC destruction are not green, because the usual approach regards it only as a waste disposal technology. The worst flaw of such approach is the generation of large volumes of waste (though non-hazardous). In fact, co-milling reagents are used in large excess respect to organic pollutant amount to ensure complete carbonization of the carbon skeleton^28^,^61^, so the final volume of the milled residue can be several tens of times the pollutant volume. The rationale of this choice is the employment of cheap reagents such as CaO and SiO_2_ in order to reduce treatment costs. However, in compliance with the “*waste prevention*” principle of Green Chemistry, more reactive and, truly, more expensive co-milling reagents, like Bi_2_O_3_ and La_2_O_3_, can be utilized to achieve full conversion at stoichiometric ratio. The reagent cost issue can be overcome by selecting appropriate chemicals and conditions to produce high added value materials such as BiOBr and LaOBr.

Accordingly, another principle that is never considered in common MC approach is the “*atom economy*”, simply because there are no products of interest. On contrary, when a valuable material is generated, this should incorporate the highest number of reagents’ atoms. The oxybromide formation reactions were conducted with unitary metal-to-bromine molar ratio, so the atom recovery for Br, Bi, and La is 100%.

An aspect that is often considered the Achilles heel of MC destruction technology is the energy efficiency. In general, industrial milling equipments are energivorous, so treatment processes that need long time milling are expensive. However, milling facilities usually work at environmental temperature and pressure, and secondary processes are very few. Therefore, cost analysis often highlights that high energy milling is a cost effective alternative. With regard to the present specific case, oxyhalides traditional production is carried out at high temperature for long time (~48 h), e.g. by direct solid state reaction between metal oxide and its halide (~1200 °C) or by thermohydrolysis (~900 °C) of metal halide under controlled humid atmosphere^62^. A brief survey of industrial furnaces’ characteristics points out that a middle-scale plant (with a capacity of 100 kg) would require ~23 kW, thus consuming 1.12 kWh energy per kg of product with a productivity of 2.1 kg h^−1^. *Per contra*, a milling facility with the same capacity, utilizing a centrifugal high energy mill (centrifugal factor 30 **g**), would necessitate of ~8.2 kW, with an energy consumption of 0.13 kWh kg^−1^ and a productivity of 6.3 kg h^−1^ (a milling time of 16 h is considered, double of the time employed in this work at laboratory-scale, because Pulverisette-7 ball mill is more energy intensive, with a centrifugal factor of 40 **g**). In this case, the HEBM itself is a cheaper solution than traditional thermal processes, so it complies with the “*energy efficiency*” principle.

In conclusion, a new approach for the MC destruction is proposed. Realizing opportune MC reactions, this technology can be not only an economically viable green process for toxic waste disposal, but also a greener way for beneficial reuse of POPs.

## Materials and methods

### Materials

HBCDD, TBBPA (Aladdin chemistry Co Ltd, China), decaBDE, and HBB (J&K Scientific Ltd, China) were used as bromine donors for the MC reaction with oxides.

Bismuth (III) oxide and lanthanum (III) oxide (Sinopharm chemical reagent Co Ltd, China) were utilized as reagents and were heated at 800 °C for 2 h prior use to eliminate adsorbed water.

All organic solvents used for the extraction and determination procedures were of HPLC grade (J.T. Baker Inc, USA), while ultra-pure water was prepared by a Milli-Q system (Millipore, USA).

### Milling experiments

HEBM was carried out employing the planetary ball mill Pulverisette 7 (Fritsch GmbH, Germany), at 700 rpm (jar-to-planetary disk speed ratio of −2), using zirconia jars (volume 45 mL) loaded with 3 g of reagent mixture and 7 zirconia balls (Ø15 mm, total weight 73.5 g). The reagent mixture was prepared with one BFR compound (viz. decaBDE, HBCDD, TBBPA, HBB) and one co-milling reagent (viz. Bi_2_O_3_, La_2_O_3_), utilizing a bromine-to-metal atomic ratio Br:(Bi,La) equals to 1. Milling time was varied to achieve the completion of the reaction and the destruction of the pollutants; intermediate times sampling was conducted.

### BFRs extraction and quantification

In order to confirm BFR destruction after HEBM treatment, each milled sample was extracted by ultrasounds and quantified by an appropriate chromatographic methodology.

Concerning DecaBDE and HBB determination, a portion of the milled residue (~0.10 g) underwent a double extraction with hexane–acetone (1:1, v/v). In the first passage, 20 mL of solvent were utilized with 15 min of ultrasonic treatment; then, the liquid phase was separated by 10 min centrifugation (1000 *g*); finally, further 30 mL solvent were employed in the second extraction step with 15 min treatment in ultrasonic bath, and the two extracts were combined and brought to the final volume of 50 mL in a volumetric flask (to compensate solvent evaporation losses). The solution was filtered by 0.45 μm polytetrafluoroethylene (PTFE) membrane and analyzed by a GC-MS (Shimadzu 2010, Japan) equipped with a DB-5HT column (15 m × 0.25 mm i.d., 0.10 μm film thickness), operated in negative ion chemical ionization (NCI) mode.

With regard to the determination of HBCDD and TBBPA, an aliquot (~0.10 g) of each milled sample was extracted with 50 mL methanol utilizing the same procedure described above. After the combination of the 2 extracts obtained from the first and the second step, and the volume adjustment in a 50 mL volumetric flask, the liquid was filtered by 0.22 μm polyethersulfone (PES) filter and then subjected to instrumental analysis. A high performance liquid chromatography (HPLC, Ultimate 3000, DIONEX Co., USA) with a C18 reversed phase column (2.1 mm × 50 mm, 3.5 μm particles, XBridge, Waters, USA) was utilized for quantification, using methanol and water by gradient elution with a flow rate of 0.3 mL/min. A tandem mass spectrometer equipped with electro-spray ionization (ESI-MS-MS, API3200, AB Sciex, USA) was employed as detector; MS–MS parameters were optimized before use, and measurements were performed in negative ion mode.

Quality assurance and quality control were conducted with the method blank and duplicate split samples. A method blank was conducted during each batch of samples to confirm the blank value had no statistical significance. The deviation of duplicate samples was within 5%.

### Products identification

In order to identify the inorganic phase products, X-ray diffraction measurement were conducted by an X-ray diffractometer (SmartLab, Rigaku, Japan) equipped with a Cu Kα radiation with 2θ  = 10–60° at speed of 10° min^−1^. Thermogravimetric analysis was performed as well, with a TG8120 analyzer (Rigaku, Japan) in nitrogen flow, using 40 mg sample and 10 °C min^−1^ heating rate from 25 °C to 600 °C.

Transformations of BFR chemical structure during milling were assessed by Fourier transform infrared spectroscopy (FTS3000, Digilab, USA) with the KBr disk method. In addition samples were characterized by Raman spectroscopy using a Labspec Raman spectrograph (HORIBA Jobin Yvon, France) with a He laser beam at the 632 nm line.

Gas phase analysis after milling was carried out by an on-line mass spectrometer (HPR-20 QIC, Hiden analytical, UK).

## Additional Information

**How to cite this article**: Cagnetta, G. *et al*. Mechanochemical conversion of brominated POPs into useful oxybromides: a greener approach. *Sci. Rep.* **6**, 28394; doi: 10.1038/srep28394 (2016).

## Supplementary Material

Supplementary Information

## Figures and Tables

**Table 1:** Atmosphere composition in the jar after HEBM.

**Oxide**	**BFR**	**Milling time (h)**	**CO**_**2**_ **(%)**	**N**_**2**_ **(%)**	**O**_**2**_ **(%)**	**Ar (%)**
Bi_2_O_3_	DecaBDE	6	47.82	40.62	11.10	0.4584
	HBCDD	8	38.76	47.80	12.91	0.5282
	TBBPA	8	17.27	64.87	17.12	0.7397
	HBB	8	16.64	64.89	17.66	0.7192
La_2_O_3_	DecaBDE	6	1.040	77.34	20.76	0.8605
	HBCDD	8	0.03440	78.31	20.79	0.8657
	TBBPA	8	0.02470	78.22	20.85	0.9053
	HBB	8	0.02370	77.80	21.28	0.9047
