# Supplementary material for: Mechanochemical conversion of brominated POPs into useful oxybromides: a greener approach
Source: Sci Rep. 2016 Jun 21;6:28394. doi: 10.1038/srep28394 (PMC4914857; doi:10.1038/srep28394)
Supplement: Supplementary Information [file srep28394-s1.doc]

**Mechanochemical conversion of brominated POPs into useful oxybromides: a greener approach**

**SUPPORTING INFORMATION**

Giovanni Cagnetta^1^, Han Liu^2^, Kunlun Zhang^1^, Jun Huang^1^,*, Bin Wang^1^, Shubo Deng^1^, Yujue Wang^1^, Gang Yu^1^

^1^State Key Joint Laboratory of Environment Simulation and Pollution Control (SKJLESPC), Beijing Key Laboratory of Emerging Organic Contaminants Control (BKLEOCC), School of Environment, POPs Research Center, Tsinghua University, Beijing 100084, P. R. China

^2^Beijing Normal University, School of Environment, 19 Xinjiekouwai St., Haidian District, Beijing 100875, P. R. China

*Corresponding author: huangjun@mail.tsinghua.edu.cn

Tab. S1 – Comparison between theoretical and experimental weight losses in TGA of milled samples

| Oxide | BFR | Milling time (h) | [BFR]/[BFR]_0_  (%) | BFR decomposition | | Oxybromide decomposition | |
| --- | --- | --- | --- | --- | --- | --- | --- |
|  |  |  |  | theoretical weight loss | experimental weight loss | theoretical weight loss | experimental weight loss |
| Bi_2_O_3_ | DecaBDE | 0 | 100 | 29.1 | 28.1 | 0.0 | 0.0 |
|  |  | 0.5 | 42 | 12.2 | 10.5 | 26.4 | 32.6 |
|  |  | 1 | 4 | 1.2 | 0.8 | 43.7 | 48.2 |
|  |  | 2 | 1 | 0.3 | 0.6 | 45.0 | 49.2 |
|  | HBCDD | 0 | 100 | 31.5 | 35.3 | 0.0 | 1.1 |
|  |  | 0.5 | 66 | 20.8 | 18.6 | 15.0 | 11.5 |
|  |  | 1 | 47 | 14.8 | 10.8 | 23.3 | 20.7 |
|  |  | 2 | 9 | 2.8 | 2.6 | 40.0 | 40.1 |
|  | TBBPA | 0 | 100 | 36.9 | 38.6 | 0.0 | 0.0 |
|  |  | 0.5 | 66 | 24.3 | 25.3 | 13.8 | 10.4 |
|  |  | 1 | 27 | 10.0 | 12.9 | 29.6 | 20.3 |
|  |  | 2 | 10 | 3.7 | 3.4 | 36.5 | 29.2 |
|  | HBB | 0 | 100 | 28.3 | 21.2 | 0.0 | 0.0 |
|  |  | 0.5 | 86 | 24.3 | 13.9 | 6.4 | 5.1 |
|  |  | 1 | 51 | 14.4 | 9.5 | 22.6 | 19.8 |
|  |  | 2 | 3 | 0.8 | 1.1 | 44.7 | 43.1 |
| La_2_O_3_ | DecaBDE | 0 | 100 | 37.1 | 37.7 | - | - |
|  |  | 1 | 51 | 18.9 | 27.4 |  |  |
|  |  | 2 | 28 | 10.4 | 17.0 |  |  |
|  |  | 4 | 1 | 0.4 | 1.5 |  |  |
|  | HBCDD | 0 | 100 | 39.6 | 37.5 | - | - |
|  |  | 2 | 63 | 25.0 | 20.0 |  |  |
|  |  | 4 | 25 | 9.9 | 8.7 |  |  |
|  |  | 8 | 2 | 0.8 | 1.7 |  |  |
|  | TBBPA | 0 | 100 | 45.5 | 25.0 | - | - |
|  |  | 2 | 64 | 29.1 | 17.8 |  |  |
|  |  | 4 | 24 | 10.9 | 5.9 |  |  |
|  |  | 8 | 1 | 0.5 | 0.5 |  |  |
|  | HBB | 0 | 100 | 36.1 | 38.5 | - | - |
|  |  | 2 | 75 | 27.1 | 32.7 |  |  |
|  |  | 4 | 39 | 14.1 | 15.5 |  |  |
|  |  | 8 | 3 | 1.1 | 1.2 |  |  |

Tab. S2 - Attribution of the absorption frequencies in FT-IR analysis (see fig. 5)

| Oxide | BFR | Frequency  (cm^-1^) | Attribution |
| --- | --- | --- | --- |
| Bi_2_O_3_ | decaBDE | 513 | Bi-O vibration mode |
|  |  | 600-800 | C-Br vibration modes |
|  |  | 966 | C-O-C bending |
|  |  | 1300-1400 | benzene ring bonds vibration modes |
|  | HBCDD | 435 | Bi-O vibration mode |
|  |  | 508 | Bi-O vibration mode |
|  |  | 600-700 | C-Br vibration modes |
|  |  | 700-1300 | alicyclic ring vibration modes |
|  |  | 1400-1500 | H-C-H bending |
|  |  | 2940 | C-H stretching |
|  | TBBPA | 500-800 | C-Br vibration modes |
|  |  | 870 | C-O stretching |
|  |  | 1000-1500 | C-H vibration modes |
|  |  | 1557 | benzene ring bonds vibration mode |
|  |  | 3515 | O-H stretching |
|  | HBB | 430 | Bi-O vibration mode |
|  |  | 510 | Bi-O vibration mode |
|  |  | 560 | C-Br stretching |
|  |  | 1200-1350 | benzene ring bonds vibration modes |
| La_2_O_3_ | decaBDE | 500 | La-O vibration mode |
|  |  | 600-800 | C-Br vibration modes |
|  |  | 966 | C-O-C bending |
|  |  | 1300-1400 | benzene ring bonds vibration modes |
|  | HBCDD | 540 | C-Br vibration mode (residual peak of the band covered by the 640 cm^-1^ peak) |
|  |  | 640 | La-OH stretching |
|  |  | 750-1300 | alicyclic ring vibration modes |
|  |  | 1400-1500 | H-C-H bending |
|  |  | 2940 | C-H stretching |
|  |  | 3610 | O-H stretching |
|  | TBBPA | 500-800 | C-Br vibration modes |
|  |  | 640 | La-OH stretching |
|  |  | 870 | C-O stretching |
|  |  | 1000-1500 | C-H vibration modes |
|  |  | 1557 | benzene ring bonds vibration mode |
|  |  | 3610 | O-H stretching |
|  | HBB | 500 | La-O stretching |
|  |  | 560 | C-Br stretching |
|  |  | 640 | La-OH stretching |
|  |  | 1200-1350 | benzene ring bonds vibration modes |
|  |  | 1380 | C-O vibration modes due to the presence of carbon (i.e. carbonate) |
|  |  | 1480 |  |
|  |  | 3610 | O-H stretching |
